# The Therapeutic Potential for Targeting Group 2 Innate Lymphoid Cells in Asthma

**DOI:** 10.3389/fimmu.2022.930862

**Published:** 2022-07-13

**Authors:** Takahiro Matsuyama, Hiromi Matsuyama, Yoichi Dotake, Koichi Takagi, Kentaro Machida, Hiromasa Inoue

**Affiliations:** Department of Pulmonary Medicine, Graduate School of Medical and Dental Sciences, Kagoshima University, Kagoshima, Japan

**Keywords:** group 2 innate lymphoid cells (ILC2s), airway inflammation, innate immune network, biologics, asthma

## Abstract

T helper type 2 cells (Th2 cells) and group 2 innate lymphoid cells (ILC2s) play an important role in the pathophysiology of asthma, including airway eosinophilic inflammation. ILC2s are activated by epithelial-derived cytokines [interleukin-25 (IL-25), IL-33, and thymic stromal lymphopoietin (TSLP)] from airway epithelial cells, leading to the release of high amounts of type 2 cytokines, such as IL-5 and IL-13. ILC2s induce airway inflammation in an antigen-independent manner, and ILC2s are considered to be involved in the pathogenesis of asthma exacerbation. Furthermore, ILC2 activation might also confer steroid resistance. Many recent studies in humans and mice are increasingly demonstrating that the function of ILC2s is regulated not just by epithelial-derived cytokines but by a variety of cytokines and mediators derived from innate immune cells. Furthermore, the biologics targeting these cytokines and/or their receptors have been shown to reduce asthma exacerbations and improve lung function and quality of life in asthmatics. This article reviews the current treatment landscape for type 2 airway inflammation in asthma and discusses the therapeutic potential for targeting ILC2s.

## Introduction

Asthma is a heterogeneous disease characterized by chronic airway inflammation, reversible airway obstruction, and airway hyperresponsiveness (AHR) (1). The prevalence of asthma is increasing in many parts of the world that have adopted aspects of the western lifestyle, and the disease poses a substantial global health and economic burden (2). Respiratory allergic diseases, including asthma, are associated with type 2 cytokines, such as interleukin-4 (IL-4), IL-5, and IL-13, which induce airway eosinophilia, mucus hyperproduction, AHR, and immunoglobulin (Ig)E class switching. There are allergic asthma phenotypes triggered by exposure to allergens and associated with allergic sensitization and adaptive immunity. There are also non-allergic asthma phenotypes triggered by exposure to environmental factors, such as air pollution, including ozone, cigarette smoke, and diesel particles; viral infection, and stress, which are associated with innate immunity independent of T helper type 2 (Th2) cells (3). Non-allergic asthma is induced by IgE-independent allergic reaction and the involvement of group 2 innate lymphoid cells (ILC2s). These different pathways and phenotypes often coexist and act in synergy in patients, and distinct pathogenic mechanisms probably underlie each of these pathways and phenotypes (3).

In 2010, ILC2s were discovered in gut-associated mucosal tissues (4–6). ILC2s do not express hematopoietic lineage markers (Lin) and respond to epithelial cell-derived cytokines, such as IL-25, IL-33, and thymic stromal lymphopoietin (TSLP). Subsequently, activated ILC2s produce high amounts of IL-5 and IL-13 (7). Since ILC2s have been recognized for their ability to drive type 2 immune responses in experimental animal models in the absence of T cells, ILC2s are a T cell-independent source of type 2 cytokines, considered to play an important role in the pathogenesis of allergic diseases including asthma (7, 8). Furthermore, many studies in both humans and mice focused on ILC2s, investigating their main features such as development from a precursor, stimuli for their activation or inhibition, their plasticity, and their classification in different subsets (9).

Pharmacotherapy for asthma is based on inhaled corticosteroid (ICS), with add-on treatments if needed, such as long-acting β_2_ agonist (LABA), leukotriene receptor antagonist (LTRA), and long-acting muscarinic antagonist (LAMA). We previously reported that LAMA suppressed innate-type-induced airway inflammation (10). This mechanism may contribute to the suppressive effect of asthma exacerbations. Approximately 5% of asthmatics have severe asthma that requires treatment with high-dose ICS plus a second controller and/or systemic corticosteroids to prevent asthma from becoming uncontrolled or that remains uncontrolled despite these therapies (11). Recently, patients with uncontrolled severe asthma have been considered candidates for biologics (12). In this review, we highlight the current treatment landscape for type 2 airway inflammation in asthma. Moreover, we discuss the therapeutic potential for targeting ILC2s (13).

## The Role of ILC2s in the Pathophysiology of Asthma

### ILC2 and Asthma Pathophysiology

In murine models, protease allergens, such as papain and house dust mites (HDM), damage the airway epithelial cell barrier *via* their proteolytic activity and release epithelial-derived cytokines, including IL-25, IL-33, and TSLP. Then, these cytokines induce ILC2 activation, leading to the production of high amounts of IL-5 and IL-13 derived from ILC2s. While T cells require priming by antigen-presenting cells upon first contact with an antigen, ILC2 activation is induced by epithelial-derived cytokines in an antigen-independent manner (14). Further, in a recent study, IL-33 and TSLP are shown to be produced from adventitial stromal cells other than airway epithelial cells. These cytokines derived from adventitial stromal cells are shown to accumulate ILC2 in regions around bronchioles and large blood vessels, where formed niches (15). ILC2s can control some of the features of asthma, such as AHR, goblet cell hyperplasia, and eosinophilia (8, 16, 17). Several groups have studied the role of human ILC2s in the pathophysiology of asthma. The number of ILC2s and the ability of cytokine production from ILC2s in peripheral blood were increased in asthmatics (18–21). Allergen exposure also increased the frequency of IL-5^+^, IL-13^+^, and prostaglandin D_2_ (PGD_2_) receptor chemoattractant receptor-homologous molecule expressed on Th2 cells-positive (CRTH2^+^) ILC2s in the sputum of asthmatics (22). Furthermore, the frequency of ILC2s in bronchoalveolar lavage fluid (BALF) also was increased in asthmatics (23). The frequency of ILC2s positively correlated with sputum and peripheral blood eosinophils and fractional exhaled nitric oxide (FeNO) levels and negatively correlated with predicted FEV_1_% (19, 24). Moreover, the numbers of total and type 2 cytokine-producing ILC2s in the blood and sputum of patients with severe asthma were significantly higher than those of mild asthmatics (20). In regard to epithelial-derived cytokines, the levels of IL-33 and TSLP were significantly increased in BALF from asthmatics. Furthermore, IL-33 levels in BALF were shown to positively correlate with the severity of asthma (23, 25). These findings suggest that ILC2s play an essential role in the pathophysiology of asthma.

### ILC2 and Virus-Induced Asthma Exacerbation

ILC2s are also considered to be involved in the pathogenesis of asthma exacerbation. Viral respiratory infections are the most common cause of asthma exacerbations in both children and adults (26). Respiratory syncytial (RS) virus infection has been shown to result in not only neutrophilic inflammation but also eosinophilic inflammation (26, 27). RS virus enhanced type 2 cytokine production and pulmonary eosinophilic infiltration in an ovalbumin-induced murine asthma model (28). In a RS virus-inoculated murine model, RS virus also induced IL-13-producing ILC2 proliferation and activation in a TSLP-dependent manner (29). Furthermore, influenza A virus infection induced AHR, independently of Th2 cells and adaptive immunity. Influenza virus infection-induced AHR required the IL-33-IL-13 axis and ILC2s (30). In studies with rhinovirus (RV) inoculation, asthmatics increased the levels of nasal IL-33 and type 2 cytokines. Furthermore, human primary bronchial epithelial cells released IL-33, and ILC2s in response to the supernatants of RV-infected bronchial epithelial cell produced type 2 cytokines (31). In another study of patients with moderate asthma inoculated with RV, ILC2 numbers in BALF were also increased during viral infection, and ILC2-mediated inflammation in asthmatics was associated with the severity of asthma (32). Thus, these results support the potential role of ILC2s in the pathogenesis of virus-induced asthma exacerbations.

### ILC2 and Steroid Resistance

TSLP is considered to promote the steroid resistance of murine and human ILC2s. In a murine model, the IL-33/ST2 (IL-33 receptor) pathways have been shown to be sensitive to steroids, and the induction of the TSLP/STAT5 pathway has been related to steroid resistance in IL-33-stimulated ILC2s (33). In human ILC2s, TSLP induced the steroid resistance in a MEK- and STAT5-dependent manner, and elevated levels of TSLP in BALF of asthmatics correlated with steroid resistance (25). Further, dexamethasone attenuated IL-5 production by the stimulation with tumor necrosis factor-like protein 1A (TL1A) or TSLP, but not by the combination of TL1A and TSLP in human ILC2s. Thus, the interaction between TL1A/death receptor 3 (DR3) pathway and TSLP may be critical for steroid resistance in human ILC2s (34). A recent study also indicated that ILC2s expressing CD45RO^+^ rather than CD45RA^+^ were associated with steroid resistance. The frequencies of CD45RO^+^ inflammatory ILC2s (iILC2s) in blood were increased in asthmatics and positively correlated with disease severity (24). These reports suggest that ILC2s might affect steroid resistance.

## The Therapeutic Potential of Targeting the Interaction Between ILC2s and Immune Cells in Asthma

### Interaction Between ILC2 and Other Immune Cells

ILC2s function as initiators of adaptive immunity or as responders to signals produced by immune cells and structured cells (35). Many studies have demonstrated that a network exists between ILC2s and immune cells or non-immune cells, and that their interaction also plays an important role in orchestrating type 2 immune responses by cell-cell contacts or by communication *via* soluble factors, such as cytokines, lipid mediators, and hormones. In regard to the association between ILC2s and T cells, IL-2 derived from T cells can promote ILC2 proliferation and IL-13 production (36). ILC2s express MHC class II, and ILC2s by themselves might be identified as antigen-presenting cells and might modulate naive T cell activation (36, 37). Furthermore, ILC2s interact with other innate effector cells other than T cells to initiate type 2 inflammation. ILC2-derived IL-13 not only triggered mucus secretion and AHR directly but also promoted dendritic cell (DC) migration to the draining lymph nodes, leading prime naive T cells to differentiate into Th2 cells (38). Additionally, ILC2-derived IL-13 is shown to induce the production of CCL17 derived from DCs, leading to the attraction of CCR4^+^ memory Th2 cells (39). Although DCs and macrophages have been known to produce TL1A to regulate the adaptive immune response by co-stimulating T cells through DR3 (40), recent studies have demonstrated that TL1A was able to directly stimulate ILC2s derived from human blood, murine mesenteric lymph nodes, and lungs to produce type 2 cytokines independent of IL-25 or IL-33 (41, 42). ILC2s also interact with granulocytes, such as basophils and mast cells, to regulate type 2 immune responses. Although eosinophils are activated by IL-5 derived from ILC2s, it has been reported that eosinophils produce IL-4 by the stimulation of IL-33 (43). Recent studies have demonstrated that basophils interacted with other immune cells and non-hematopoietic cells through cell-to-cell contact or cytokines and proteases, affecting the regulation of immune and allergic responses (44). In regard to the association with ILC2, basophils have been shown to trigger ILC2 proliferation and activation *via* basophil-producing IL-4 (45, 46). In the study of murine lungs, basophil-producing IL-4 had an important role in ILC2-derived type 2 cytokine production, subsequently leading to protease-induced airway eosinophilic inflammation (45). In regard to the interaction between ILC2s and mast cells, mast cell-derived IL-2 enhances the expansion of regulatory T cell (Treg) numbers by IL-33 stimulation, thereby suppressing ILC2-dependent allergic inflammation in a murine model. In this study, mast cells suppress protease-induced airway eosinophilic inflammation through a mast cell-IL-2-Treg-IL-10-ILC2 axis (47).

### Therapeutic Potential of Targeting Cells Interacting With ILC2 in Asthma

In asthma, recent studies have indicated the therapeutic potential for targeting the crosstalk between ILC2s and other immune cells. Our group revealed that tiotropium, LAMA, attenuated ILC2-dependent airway eosinophilic inflammation by suppressing IL-4 production from basophils and subsequently regulating ILC2 activation in a murine model (10). Tiotropium is mainly attributed to muscarinic M3 receptor (M3R) inhibition. However, ILC2s hardly expressed M3R. *In vitro* study, tiotropium did not affect IL-33-induced IL-5 and IL-13 production from ILC2s, suggesting that tiotropium regulated ILC2s indirectly. In contract, basophils exhibited higher expression of M3R as compared with that of other immune cells. Tiotropium suppressed IL-33-induced IL-4 production from basophils. Moreover, in co-culture of basophils and ILC2s, the production of IL-5 and IL-13 was decreased by tiotropium. Furthermore, M3R was shown to be expressed on human basophils. Tiotropium also reduced the production of human basophil-derived IL-4 (10). Because the addition of tiotropium to ICS and LABA has been reported to reduce the frequency of asthma exacerbation in patients with severe asthma (48), the inhibitory effects of LAMA on ILC2-mediated airway inflammation may contribute to reducing the risk of asthma exacerbation (10). Toll-like receptors (TLRs) recognize components of viruses and bacteria to trigger an innate immune response. R848, a TLR7 agonist, stimulated IL-33-induced interstitial macrophages (IMs) to attenuate ILC2-mediated airway eosinophilic inflammation through IM-derived IL-27 (49), which suppressed the proliferation and type 2 cytokine production of murine and human ILC2s (50, 51). Other TLR7 agonists, AZD8848 and GSK2245035, have been evaluated as therapeutic agents in clinical trials for asthma. However, the effect of AZD8848 on the fall in FEV_1_ of late asthmatic response after allergen challenge was not sustained for 4 weeks, and GSK2245035 also did not show clinical improvement (52, 53). ILC2-mediated airway inflammation has been associated with allergen-independent stimuli such as viral infection. Thus, TLR7 agonists, as well as LAMA, are expected to have an effect on regulating asthma exacerbation, especially those associated with viral infection. In these results, LAMA and TLR7 agonists are considered to act on the innate immune network, leading to the indirect inhibition of ILC2-dependent airway inflammation *via* basophils and macrophages (**Table 1**).

**Table 1:** Summary of drugs targeting cells interacting with ILC2 in murine model.

Drugs	Targets	Target cell	Effect on airway inflammation			
			BALF eosinophils	BALF ILC2s	Cellular infiltration around airway	Mucus secretion
Tiotropium	M3R	Basophils	↓	↓	↓	↓
R848	TLR7	Interstitial macrophages	↓	↓	↓	↓

BALF, bronchoalveolar lavage fluid; ILC2, group 2 innate lymphoid cell; M3R, muscarinic M3 receptor; TLR7, toll-like receptor 7.

### Neuropeptides, Lipid Mediators, Sex Hormones, and Angiotensin II

In addition to cytokines, various factors, including lipid mediators, neuropeptides, sex hormones, also regulate ILC2s and are directly involved in airway inflammation. Lipid mediators are produced by various cells including immune cells, such as neutrophils, mast cells, basophils and macrophages (54, 55). However, the major source of lipid mediators acting on ILC2 has not been well understood. Cysteinyl leukotriene receptors (CysLTR) are expressed on ILC2. In an *Alternaria* species-induced murine model, leukotriene D_4_ (LTD_4_) activates ILC2 to rapidly produce high levels of IL-4, IL-5, and IL-13 (56). Further, PGD_2_, which are produced by mast cells, causes human and mouse ILC2 chemotaxis and type 2 cytokine production *via* CRTH2 in the lungs (22, 57, 58). In the IL-33-induced murine model, PGE_2_ results in decreased eosinophils numbers and type 2 cytokine production, and ameliorated ILC2-mediated lung inflammation (59). In the study of mouse and human ILC2s, PGE_2_ has a suppressive effect on IL-33-induced ILC2 proliferation and type 2 cytokine production *in vitro* (59, 60). PGI_2_ also has an inhibitory effect on ILC2s. In an *Alternaria* species-induced murine model, PGI_2_ suppresses IL-5 and IL-13 protein expression, and IL-5- and IL-13-expressing ILC2 numbers in lungs. Moreover, in the study of human ILC2 stimulated with IL-2 and IL-33, PGI_2_ inhibits IL-5 and IL-13 production (61). Regarding neuropeptides, ILC2s express neuromedin U receptor 1 (NMUR1) and calcitonin gene-related peptide (CGRP) receptor (CALCRL and RAMP1) (62, 63). Neuromedin U is expressed on the neurons in the thoracic dorsal root ganglia, which contain afferent sensory neurons (62), and CGRP is produced by pulmonary neuroendocrine cells (PNECs) that reside near ILC2s at airway branch points (63). Neuromedin U and CGRP induce ILC2-mediated allergic lung inflammation. ILC2s also express α7nAChR, a nicotinic acetylcholine receptor. Acetylcholine including nicotine is derived from parasympathetic nerves (64). GTS-21, an α7nAChR agonist, is associated with reduced airway inflammation and AHR in the IL-33-induced murine model (65). Sex hormones such as androgen regulate the development and function of ILC2s. ILC2 progenitors (ILC2P) express androgen receptors. In male mice, HDM or IL-33-induced airway inflammation is less prominent than those in female mice (66). Further, ILC2 numbers in the blood of asthmatics are higher in women compared to men (67). In a recent study, ILC2s are shown to express AT1a, angiotensin II receptor. Angiotensinogen, renin, and angiotensin-converting enzyme (ACE), which are the enzymes essential for angiotensin II production, are expressed on airway epithelial cells (68). Thus, angiotensin II may be derived from airway epithelial cells. Angiotensin II induces ILC2 responses in a cell-intrinsic and IL-33-dependent manner, leading to airway inflammation. Furthermore, the levels of angiotensin II in plasma positively correlate with the abundance of blood ILC2s and disease severity in asthmatics (68). These findings suggest that neuropeptides, lipid mediators, sex hormones, and angiotensin II and their receptors might be potential therapeutic targets for asthma and ILC2-related diseases.

## The Efficacy of Biologics and the Potential of ILC2-Targeted Therapy in Asthma

Biologic therapies target specific inflammatory pathways involved in the pathogenesis of asthma, particularly in patients with an endotype caused by type 2 inflammation (12). These therapies have been required in patients with uncontrolled severe asthma despite optimal treatment. Currently, available biologics compose anti-IgE, anti-IL-5, anti-IL-5 receptor α (IL-5Rα), and anti-IL-4 receptor α (IL-4Rα). These biologics reduce asthma exacerbation rates and improve asthma symptoms and lung function. Additionally, randomized controlled trials have recently shown the efficacy of biologics for targeting epithelial-derived cytokines, such as TSLP and IL-33, in patients with severe asthma (**Table 2**).

**Table 2:** Summary of approved and new potential biologics for severe asthma.

Biologics	Targets	Effect on inflammatory biomarker			Effect on asthma control				OCS-sparing effect
		FeNO	Blood eosinophils	Sputum eosinophils	Exacerbation	Symptom	QOL	FEV_1_	
**Approved biologics**									
Omalizumab	IgE	↓	↓	↓	↓	↓	↑	↑	No data on RCT
Mepolizumab	IL-5	→	↓	↓	↓	↓	↑	↑	50% reduction
Reslizumab	IL-5	No data	↓	↓	↓	↓	↑	↑	No data on RCT
Benralizumab	IL-5Rα	No data	↓	↓	↓	↓	↑	↑	75% reduction
Dupilumab	IL-4Rα	↓	↑	No data	↓	↓	↑	↑	70% reduction
**New potential biologics**									
Tezepelumab	TSLP	↓	↓	↓	↓	↓	↑	↑	No significant difference ^#^
Itepekimab	IL-33	↓	↓	No data	No data	↓	↑	↑	No data
Astegolimab	ST2	→	↓	No data	↓	→	↑	→	No data

OCS, oral corticosteroid; FeNO, fractional exhaled nitric oxide; QOL, quality of life; RCT, randomized controlled trial, ^#^, unpublished data.

### Anti-IgE

Omalizumab, a humanized anti-IgE monoclonal antibody (mAb), prevents IgE from binding to high-affinity IgE receptors (FcϵRI) on mast cells and basophils, which inhibits the release of proinflammatory mediators, such as histamine and leukotrienes. Clinical trials have demonstrated that omalizumab reduces asthma symptoms and the frequency of asthma exacerbation (69, 70). The three biomarkers—FeNO, peripheral blood eosinophil counts, and serum periostin—were associated with the risk of asthma exacerbation (71). However, in asthma patients, the frequencies of ILC2s and ILC2-derived cytokine production were not influenced by omalizumab (72).

### Anti-IL-5/IL-5Rα

Mepolizumab, a humanized anti-IL-5 mAb, binds to IL-5, preventing IL-5 from binding to its receptors on eosinophils (12). Among patients with uncontrolled eosinophilic asthma who have increased sputum or peripheral blood eosinophil counts, mepolizumab has been shown to reduce the frequency of asthma exacerbation, improve lung function and asthma control, and reduce the oral corticosteroid (OCS) dose (73–75). In the study compared treatment responses of weight-adjusted intravenous reslizumab, another humanized anti-IL-5 mAb, among patients previously treated with subcutaneous mepolizumab, the blood ILC2 numbers were significantly but modestly decreased. However, ILC2 numbers in blood or sputum were not decreased by reslizumab (76). On the other hand, benralizumab, a humanized anti-IL-5Rα mAb, prevents IL-5 from binding to IL-5Rα on eosinophils. The clinical trial revealed that benralizumab had the same effect as mepolizumab in asthmatics with elevated peripheral blood eosinophil counts (77–79). Additionally, in patients with severe steroid-dependent asthma, benralizumab reduced the blood IL-5Rα^+^ ILC2 numbers but not the numbers of total ILC2s (80).

### Anti-IL-4Rα

Dupilumab, a humanized anti-IL-4Rα mAb, inhibits the signaling of both IL-4 and IL-13 by binding to IL-4Rα. Dupilumab has been shown to reduce the rate of asthma exacerbation and improve lung function and asthma control in patients with moderate-to-severe uncontrolled asthma (81, 82). Among patients with a peripheral blood eosinophil count ≥300/ µl or FeNO ≥50 ppb, the effect of dupilumab on the asthma exacerbation rate was greater (82). Moreover, dupilumab had an OCS-sparing effect while decreasing the asthma exacerbation rate and improving lung function (83). In a murine model, IL-4 might promote cytokine production from ILC2s through IL-4R (45, 46). In the study of human ILC2s cultured with IL-2, IL-7, IL-25, and IL-33, the addition of IL-4 to the ILC2s also further enhanced the production of IL-5 and IL-13 *in vitro* (84). Treatment with dupilumab reduced ILC2 numbers in the peripheral blood of asthmatics. Furthermore, dupilumab repressed the expression of IL-5 and IL-13 mRNA in ILC2s. Thus, these results demonstrate that the blockade of IL-4Rα by dupilumab suppressed ILC2 response directly or indirectly in asthmatics and might be involved in the reduced frequency of asthma exacerbation (84). Human ILC2s have been shown to express IL-4Rα (85), and dupilumab can be a novel treatment targeting ILC2s.

### Anti-TSLP

Recently, biologics targeting upstream type 2 inflammation have been developed, including anti-TSLP, anti-IL-33, and anti-ST2 therapies in addition to dupilumab. Tezepelumab, a humanized anti-TSLP mAb, prevents TSLP from interacting with its heterodimeric receptor. In a phase 2 study, tezepelumab regulated the rate of asthma exacerbation in patients with moderate-to-severe uncontrolled asthma (86). A recent study also demonstrated that tezepelumab suppressed type 2 inflammatory biomarkers, such as blood eosinophil counts, FeNO, serum IgE, type 2 cytokines, periostin, thymus and activation-regulated chemokine (TARC); there were positive correlations between these biomarkers at the baseline (87). Further, tezepelumab’s suppression of asthma exacerbation was observed regardless of blood eosinophil counts (86). In a phase 3 study involving severe uncontrolled asthmatics, tezepelumab not only reduced the frequency of asthma exacerbation but also improved lung function and asthma control, regardless of blood eosinophil counts (88). So far, available biologics have not been shown to suppress asthma exacerbations in asthmatics with type 2-low endotype. Therefore, tezepelumab is expected to have an effect on asthmatics with type 2-low endotype.

### Anti-IL-33/ST2

Genome-wide association studies have indicated that IL-33 and IL1RL1 (encoding ST2), which have obvious links to ILC2 biology, are associated with asthma susceptibility (89–91). Itepekimab is a human IgG4P mAb against IL-33. In a phase 2 trial involving patients with moderate-to-severe asthma, itepekimab led to improved lung function and fewer events indicating a loss of asthma control. Further, itepekimab also reduced blood eosinophil counts and FeNO levels (92). Astegolimab is a human IgG2 mAb that blocks IL-33 signaling by targeting ST2. In a phase 2b study involving patients with severe asthma, astegolimab suppressed annualized asthma exacerbation rates. Furthermore, the reduction of the asthma exacerbation rate for patients with low blood eosinophil counts was also comparable to the reduction in the overall population. In regard to biomarkers, astegolimab reduced blood eosinophil counts but not FeNO levels (93). ILC2-expressed IL-4R, ST2, and TSLPR; and IL-4, IL-33, and TSLP are required for ILC2 activation and expansion, as described above. Thus, suppressing these pathways that induce ILC2 responses might lead to improved asthma control.

## Concluding Remarks

**Figure 1:**
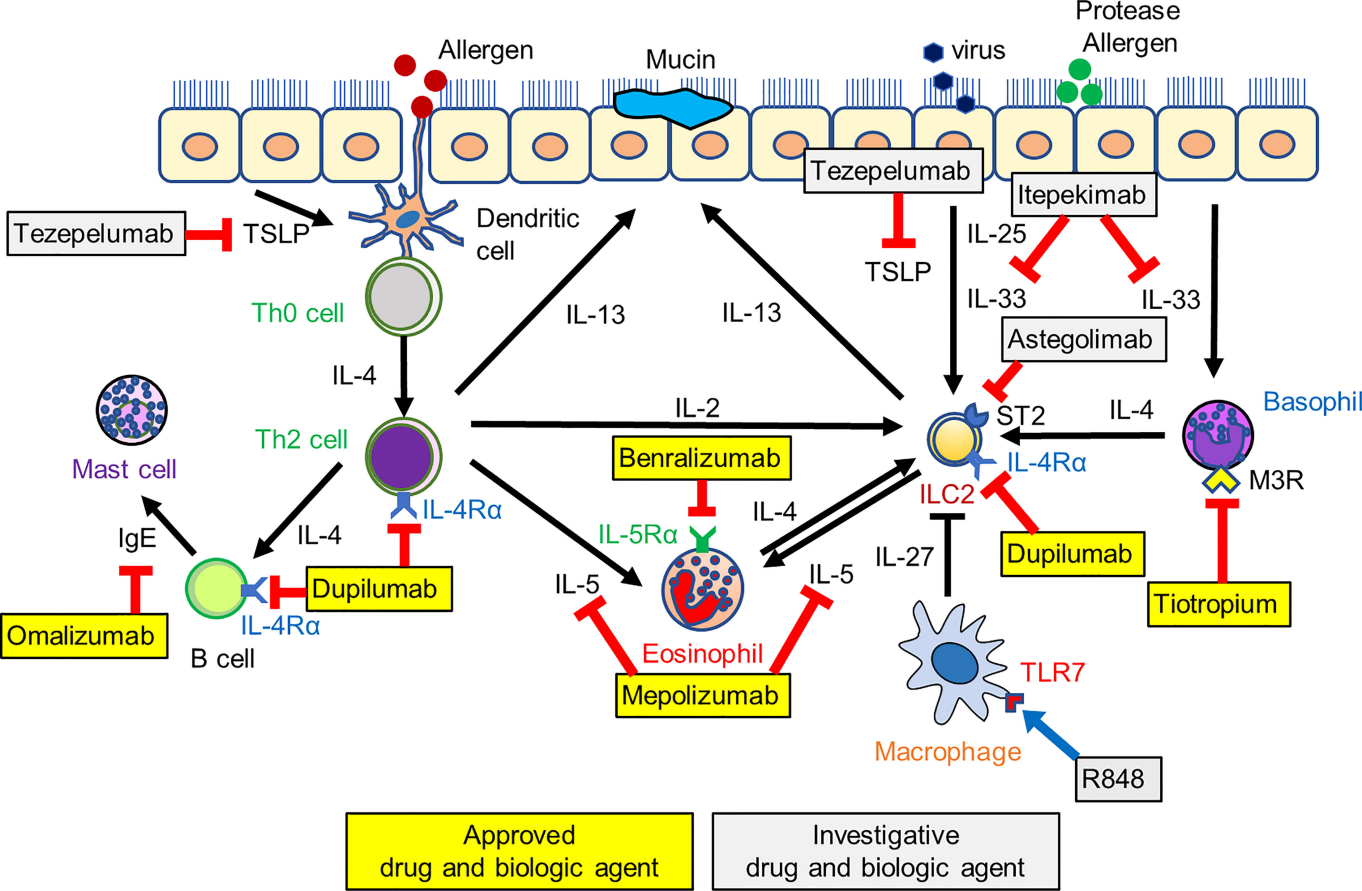
The network between ILC2s and cells of the innate and adaptive immune system and a schematic of investigative and approved biologic therapies. Biologics target IgE, IL-5 and its receptor, IL-4 receptor, and alarmins such as TSLP and IL-33, leading to the suppression of asthma exacerbation and improved asthma control. Tiotropium suppresses basophil-derived IL-4 production, and R848, a toll-like receptor7 (TLR7) agonist, induces macrophage-derived IL-27 production. These pathways also indirectly induce the suppression of ILC2-mediated airway eosinophilic inflammation. ILC2, type 2 innate lymphoid cell; M3R, muscarinic M3 receptor.

This review has highlighted current asthma treatment strategies for type 2 airway inflammation and described the therapeutic potential for targeting ILC2s (**Figure 1**). In recent studies, various pathways have been identified for regulating ILC2 function, and the network between ILC2s and the cells of the innate and adaptive immune system has been characterized. Some therapeutic agents, including tiotropium, attenuate ILC2-mediated airway inflammation indirectly by inhibiting cytokines derived from various immune cells. The regulation of the ILC2-mediated innate immune network also has an essential role in asthma control and can be linked to the potential therapeutic target.

Currently available biologics reduce asthma exacerbation and improve asthma control by blocking specific type 2 cytokines directly. However, these fail to prevent asthma exacerbation completely. This may be because the biologics inhibit pathways downstream in an immunological cascade, while leaving other cascades still active. On the other hand, tezepelumab blocks the TSLP pathway alone, leading to the sufficient suppression of type 2 inflammation. In addition to tezepelumab, itepekimab and astegolimab, which inhibit “alarmins,” also have a positive effect on asthma outcomes. Therefore, blocking alarmins, which are upstream mediators, inhibits broad type 2 inflammatory response. The observation that ILC2s are activated by alarmins suggests that ILC2-targeted therapies are expected to be effective in the initial phase of airway inflammation. However, the association between biologics and ILC2s is limited. As IL-4Rα is expressed on ILC2, dupilumab has been shown to reduce not only type 2 inflammatory biomarkers (e.g., type 2 cytokines and FeNO) but also ILC2 numbers. There are no data that tezepelumab, itepekimab, and astegolimab reduce ILC2 numbers or ILC2-derived type 2 cytokine production. Therefore, further studies are required to clarify the effects of alarmin-targeted biologics on ILC2s. These studies will be relevant for the further development of therapeutic agents for targeting ILC2s in asthma.

## Author Contributions

TM and HI wrote this manuscript. TM and KM designed the figure. HM, YD, and KT reviewed the manuscript and provided editorial input. All authors reviewed and approved the final version of the manuscript for publication.

## Funding

This work received no specific grant from any funding agency in the public, commercial, or not-for-profit sectors.

## Conflict of Interest

HI reports grants from Boehringer Ingelheim, Novartis and GlaxoSmithKline unrelated to this study; and personal fees from AstraZeneca, Boehringer Ingelheim, GlaxoSmithKline, Kyorin, and Sanofi.

The remaining author declares that the research was conducted in the absence of any commercial or financial relationships that could be construed as a potential conflict of interest.

## Publisher’s Note

All claims expressed in this article are solely those of the authors and do not necessarily represent those of their affiliated organizations, or those of the publisher, the editors and the reviewers. Any product that may be evaluated in this article, or claim that may be made by its manufacturer, is not guaranteed or endorsed by the publisher.
